# Case of Severe Chorea

**Published:** 1853-07

**Authors:** 

**Affiliations:** Medical Registrar to St. George’s Hospital


*Case of Severe Chorea.* By Dr. Barclay, Medical Registrar to St. George's Hospital.—A very severe case of this disease has just been brought to a successful termination, some few particulars of which may be found worthy a place in the *Medical Times and Gazette.* For some days the issue in life or in death was exceedingly doubtful; and the means employed, with beneficial results, being somewhat unusual in the treatment of this disease, available suggestions may be derived from their detail to guide others in the management of similar instances.

The two principal remedies used at the time when the disease took a favourable turn, and to which we are, therefore, disposed to look as those by which its progress was checked, were the inhalation of chloroform and the employment of large suppositories of quinine. The former agent now holds the first place in temporarily allaying nervous irritability and suspending muscular action; but its effects are also not merely temporary; sleep commonly follows on recovery from the immediate stupefaction; and in how many instances may not the physician say, "if he sleep he shall do well!" But it is not the troubled dream of narcotism or *quasi* intoxication that fulfils the function of "Nature's soft nurse." If natural sleep does not follow on the cessation of the artificial dose,—if the drug, whatever it may be, does not serve merely as the first provocative of a "balmy sleep," which is prolonged without artificial aid, the object of the physician will be frustrated, and the patient will awake unrefreshed by his slumbers, and, perhaps, not uninjured by the congestion of the brain which always attends in greater or less degree the action of narcotics. On this circumstance depends the very varying success which has attended the use of chloroform in states of extreme nervous irritability—mania, delirium tremens, and chorea; in each of which it has been employed at times with apparent advantage, while at others its effect has been null, or even, perhaps, pernicious. It is true that, as its effects are more transient, at the same time that they are more decided than those of any other narcotic, it may be ventured on in those doubtful cases bordering on inflammation, where, in all probability, a certain degree of congestion already exists, and where we should not feel justified in giving opium or even morphia experimentally, and, therefore, there is less limit to the cases in which its effects may be tried; yet while we know that we can for a time allay pain, annul consciousness, and calm irritability, we cannot be certain that we shall succeed in obtaining a natural sleep, and, therefore, can be by no means certain, even when no congestion exists, that its action will be curative.

Along with the calm of the narcotic, and, in fact, to give full force to its action, it was necessary to support the system, and restore its exhausted energies, by the administration of a tonic. Large doses of quinine have of late been so frequently given, with alleged benefit in some instances, and apparently without prejudice in all, that nothing need be said except with reference to the mode of administration, which was here in the form of suppository: tonics and sedatives, taken into the stomach, had failed of quieting, and restoring disordered function; and, in such cases, it always demands consideration, whether perverted assimilation may not prevent their entering the circulation in such a manner as to produce their normal effects on the system. Dr. Wilson is constantly in the habit of noticing this circumstance in the depraved states of stomach of habitual drunkards, and attempting to reach the nervous system by another channel, which may not offer the same obstruction to its absorption. With this view, opium, in the form of suppository, is frequently ordered in delirium tremens with the best effects.

The consideration, too, of the absence of the menstrual flux, the evidence of periodical congestion of the generative organs, was not without its importance in determining this application of the remedy. The catamenia had been regular up to that time, and the decided exacerbations of the symptoms occurred just when their return was expected. Two or three days had passed without any appearance of the sort, and the symptoms were gradually increasing in severity when this remedy was employed. It may be remarked, however, that, up to the period of her convalescence, the function was not restored.

Susan H—, aged 16, was admitted into St. George's Hospital, under the care of Dr. Wilson, on February 23, 1853. She had been in service, and had been much worried by illness in the house, and over-work. No other cause was known for the occurrence of the symptoms of chorea, which had been gradually supervening during a fortnight or three weeks before her admission. She had had no rheumatism, and the heart was healthy. When received into the hospital, the spasmodic movements were not by any means violent, nor very constant; but there was great distortion of feature, especially when she attempted to speak. She was ordered a strong purgative, and then directed to have six minims of Fowler's solution in pimento water, three times a day. The bowels were rather loaded, and much inclined to be costive, and the purgative was repeated on two or three occasions. She continued taking the arsenical solution until the 11th of March, and seemed, under its influeuce, to be gradually becoming quieter and steadier. She was rather out of sorts that day, and the arsenic was left off for a day or two.

The spasmodic movements now began to be more frequent and severe, and she was obliged to lie in bed. The sesquioxide of iron was given in doses of two scruples three times a day without benefit. Her nights became disturbed, and she used to scream out a good deal during the day. Seclusion had rather a calming effect upon her; and the bed was screened off from the rest of the ward, during which she used to have occasional short slumbers; but, for the most part, she was very restless; the expression of her face was rather wild, and the spasmodic movements constant and severe.

On the 17th, the bowels having been well purged, she was ordered to have a third of a grain of tartar emetic every three hours, and half a grain of acetate of morphia at night. This produced no sickness, and seemed partially to calm her; it was continued a second day, at intervals of four hours. She had no sleep that night, and was evidently no better; without being very violently tossed about, there was excessive restlessness and desire of movement; her face was much distorted, and the expression wild, and almost maniacal; and, as she sat upon her bed, with her legs drawn up under her, and her hair in wild confusion about her face, she looked much more like a proper inmate for Bedlam, than a person suffering simply from chorea, and the more so, because of her constant cries; but the nurse satisfied herself that she was free from delirium, and perfectly conscious on all occasions.

She had now passed the catamenial period, with no return of the menstrual flux. Her nights were sleepless, and a quarter of a grain of morphia was given every three hours to six times, but without benefit. She seemed to be gradually becoming exhausted by the constant jactitation and want of sleep. Wine had been given; yet the tongue was becoming dry and brown, the lips covered with sordes, and the pulse quick and feeble. Her aspect was distressed, and she was tossed about very violently by the spasmodic movements of the disorder.

On the 21st she was directed to inhale chloroform, and to have a suppository, with two grains of opium at once, and a draught with four grains of camphor, and some chloric ether every two hours. The first inhalation quieted her, but she awoke almost immediately after the first effects had passed off; it was not repeated till night, and, in the mean time, eight grains of quinine in suppository were ordered every hour for six times. She had some sleep after the second inhalation, and it was repeated the following forenoon. She was decidedly quieter, and the draughts and the suppositories, which had not been all given with perfect exactness, although very frequently during the night, were continued at intervals of four hours. The chloroform inhalation was again repeated on the evening of the 22d. She slept pretty well after it through the night, and fell asleep naturally next morning, waking up to have her dinner, and almost immediately going to sleep again, and continuing to dose all day. From this time she continued to improve rapidly. She was still a little odd-looking in the face, and restless jactitations lasted for some time; but she gradually became steadier. On the 4th April she was ordered Griffith's mixture, and on the 15th was discharged recovered.—*London Medical Times and Gazette.*

